# First Report and Pathogenicity Analysis of *Photobacterium damselae* subsp. *piscicida* in Cage-Cultured Black Rockfish (*Sebastes schlegelii*) Associated with Skin Ulcers

**DOI:** 10.3390/microorganisms13020441

**Published:** 2025-02-17

**Authors:** Dandan Zhou, Binzhe Zhang, Yulie Qiu, Xuepeng Li, Jian Zhang

**Affiliations:** 1 School of Ocean, Yantai University, Yantai 264005, China; 2 Shandong Engineering Research Center of Healthy Land-Sea Relay Farming of Economic Fish, Yantai 264005, China; 3 Yantai Engineering Research Center of Deep-Sea Aquaculture of Economic Fish, Yantai 264005, China

**Keywords:** *Sebastes schlegelii*, *Photobacterium damselae* subsp. *piscicida*, antimicrobial-resistant, extracellular products, pathogenicity

## Abstract

*Photobacterium damselae* subsp. *Piscicida* (PDP), a marine bacterium, has been reported to infect a variety of economically important marine species worldwide. Understanding the occurrence and pathogenicity of PDP is crucial for effective disease control and ensuring the success of aquaculture operations. In late August 2023, an epidemic outbreak of *P. damselae* subsp. *piscicida* DQ-SS1, accompanied by significant mortality, was recorded in cage-cultured black rockfish *(Sebastes schlegelii*) located on Daqin Island for the first time. Genomic analysis revealed that DQ-SS1 possesses 2 chromosomes, with a total size of 4,510,445 bp and 3923 predicted CDSs. Pathogenic genes analysis identified 573 and 314 genes related to pathogen–host interactions and virulence, respectively. Additionally, DQ-SS1 displayed susceptibility to 15 antimicrobials, was resistant to 11 antimicrobials, and was intermediately sensitive to four antibiotics. Meanwhile, the in vitro assay revealed that the extracellular products (ECP) of DQ-SS1 were lethal to macrophages and exhibited hemolysin, lipase, and amylase activities. Moreover, DQ-SS1 also demonstrated the ability to survive in fish serum and resist complement-mediated killing. The in vivo assay showed that the infected fish exhibited severe histopathological alterations, such as the infiltration of inflammatory cells, cellular degeneration and necrosis, and loose cell aggregation. Lastly, the in vivo infection assays revealed the LD_50_ of DQ-SS1 was 1.7 × 10^3^ CFU/g. This is the first study to elucidate the pathogenicity and genomic characteristics of multidrug-resistant PDP in cage-cultured *S. schlegelii*, which contributes to the advancement of diagnostic and preventative strategies for this disease in marine-cultured fishes and provides information for an in-depth study of the pathogenic mechanism of PDP.

## 1. Introduction

*Sebastes schlegelii* (Black rockfish), a typical viviparous teleost, is a member of the *Scorpaenidae* family [1]. *S. schlegelii* is popular for its high nutritional content and economic and ecological values. Meanwhile, some characteristics, such as the fast growth rate and tolerance to low water temperature, make *S. schlegelii* suitable for large-scale aquaculture [2,3]. At present, *S. schlegelii* has emerged as one of the most important mariculture species on the north-east coast of China, Korea, and Japan [4,5,6]. In Shandong Province, the production of cultured *S. schlegelii* has surpassed 10,000 tons and continues to increase annually. In recent years, due to the elevated culture density and the degradation of water quality in cage-cultured *S. schlegelii*, various pathogens, encompassing *Aeromonas salmonicida* [7], *Lactococcus garvieae* [8], *Mycobacterium marinum* [9], and lymphocystis [10], have resulted in severe mortalities and production losses.

*Photobacterium damselae*, a member of the genus *Photobacterium* of the family *Vibrionaceae*, comprises two subspecies, namely *P. damselae* subsp. *damselae* (PDD) and *P. damselae* subsp. *piscicida* (PDP) [11]. The former is an opportunistic pathogen that causes infection in a wide range of marine animals and humans [12]. The latter, previously referred to as *Pasteurella piscicida* or *Vibrio damsel*, was initially isolated from natural populations of white perch and striped bass in 1963 in Chesapeake Bay (USA) [13]. It is the primary pathogen responsible for pasteurellosis, whose incidence is associated with high temperatures, low salinity, and poor water quality [14]. PDP is recognized as one of the most significant septicemic diseases worldwide due to its high mortality rate, broad host range, and widespread distribution [14]. So far, PDP was reported to affect a diverse range of economically important fish species, including tongue sole (*Cynoglossus semilaevis*), cobia (*Rachycentron canadum*), and golden pompano (*Trachinotus ovatus*) in China [15,16,17], gilthead sea bream (*Sparus aurata*) and sea bass (*Dicentrarchus labrax*) in Europe [18,19], striped bass (*Morone saxatilis*) and white perch (*Morone americana*) in the USA [20,21], and yellowtail (*Seriola quinqueradiata*) in Japan [22]. The clinical manifestations of PDP infections vary among fish species, with some exhibiting hemorrhages on the basal fin and ulcers on the skin [15], whereas others show gray and white nodules on the internal organs [16,17].

Elucidating the occurrence and pathogenicity of a pathogen is essential for its prevention and treatment [14]. To date, information on the pathogenicity of PDP in different fish species under natural or experimental conditions is scarce. Numerous studies have revealed that extracellular products (ECPs) are also implicated in the virulence of PDP. For instance, AIP56 is a plasmid-encoded virulence factor abundantly secreted by virulent strains of PDP and responsible for the apoptosis of macrophages and neutrophils [18,19]. Similarly, Peptidoglycan hydrolase (PnpA) is a secreted protein that degrades the peptidoglycan of the host to facilitate infection [20]. In addition to these two type II secretion system (T2SS)-secreted virulence factors, a T3SS has also been identified in PDP [21,22]. Notably, PDP also possesses virulence factors for host iron acquisition [23], reducing complement-related activity [24] and superoxide radical production [25].

To the best of our knowledge, there have been no reports of PDP infection in *S. schlegelii*. This study aims to reveal the virulence of PDP in cage-cultured *S. schlegelii* and to further elucidate the pathogenic characteristics, virulence factors, and drug-resistant spectrum of PDP DQ-1. For this purpose, a comprehensive analysis was conducted encompassing the examination of physiological and biochemical characteristics, genomic sequencing, drug resistance, ECPs, histopathology, and the virulence of strain DQ-SS1.

## 2. Materials and Methods

### 2.1. Ethics Statement

Live animal experiments were performed in accordance with the guidelines of “Regulations for the Administration of Affairs Concerning Experimental Animals” promulgated by Shandong Province. Experiments involving live animals were approved by the Ethics Committee of Yantai University, with the ethical approval code No. 20230902.

### 2.2. Sampling and Bacterial Isolation

**Figure 1:**
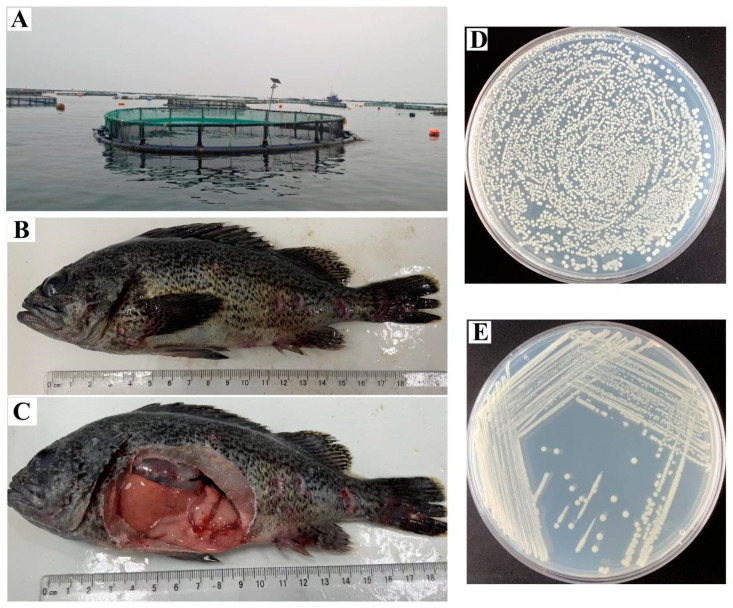
Clinical manifestations of natural infection in *Sebastes schlegelii*. (**A**) Offshore fish cage used for *S. schlegelii* rearing. (**B**,**C**) Clinical symptoms of natural DQ-SS1 infection in *S. schlegelii*. (**D**,**E**) Isolation and purification of strain DQ-SS1.

In August 2023, a sudden outbreak of disease with notable mortality was documented in cage-cultured *S. schlegelii* (about 450 g) located on Daqin Island, Yantai, China (Figure 1A). Spleen and kidney tissues were harvested from diseased fish under aseptic conditions for pathogen isolation. These tissues were homogenized in PBS and streaked onto 2216E agar plates (HopeBio, Qingdao, China), then incubated at 28 °C for 24 h. The dominant bacterial isolate was purified by repeated streaking onto marine 2216E agar plates three times and named DQ-SS1.

### 2.3. Molecular Characterization and Phylogenetic Analysis

For genomic and 16S rDNA sequencing, strain DQ-SS1 was cultured, and its DNA was extracted with a bacterial DNA extraction kit (Tiangen, Beijing, China). The concentration of the purified bacterial DNA was quantified using a Nano 300 spectrophotometer (ALLSHENG, Hangzhou, China). The bacterial 16S rRNA gene was amplified by PCR using the universal primers 27F and 1492R [26]. DNA sequencing was carried out by Sangon Biotech (Shanghai, China), and the 16S rRNA gene sequence underwent analysis via EzBioCloud (http://eztaxon-e.ezbiocloud.net/, accessed on 9 December 2023) [27] and the National Center for Biotechnology Information (NCBI). Using the Mega X software package, a phylogenetic tree was developed with the neighbor-joining method under the default parameters [28], and its stability was assessed through bootstrap analysis with 1000 replications.

### 2.4. Biochemical and Antimicrobial Resistance Characterization

The biochemical characteristics of DQ-SS1 were determined using API 20NE, API 50CH, and API ZYM strips (bioMérieux, Marcy-l’Étoile, France) following the manufacturer’s instructions. All API tests were performed in triplicate. To measure oxidase activity, an oxidase reagent (Haibo, Qingdao, China) was used, and catalase activity was evaluated by the appearance of bubbles in a 3% (*v*/*v*) H_2_O_2_ solution. The antimicrobial sensitivity test for DQ-SS1 was performed using the standard Kirby–Bauer disk diffusion method [29]. In brief, a bacterial suspension with a 0.5 McFarland standard (about 1 × 10^8^ CFU/mL) was prepared and evenly applied to the surface of 2216E agar plates, following which commercial antibiotic disks (Hangwei, Hangzhou, China) were applied to the plates. To ensure the accuracy of the test, a quality control strain, *Escherichia coli* ATCC 25922, was also analyzed in parallel. The antibiotics used in this study are listed in Table 1. After 24 h of incubation at 28 °C, the diameter of the zone around the disc was measured and the bacteria were classified as susceptible (S), intermediate (I), or resistant (R) to a particular antimicrobial agent according to the criteria set by the clinical and laboratory standards institute (CLSI). These assays were performed in duplicate.

### 2.5. Genomic Sequencing and Analysis

The genomic sequencing of strain DQ-SS1 was performed using Nanopore PromethION (Oxford Naopore Technologies, Oxford, UK) and Illumina NovaSeq PE150 (Illumina, Santiago, CA, USA) by Novogene Biotechnology Co. (Beijing, China). High-quality PacBio reads (>6000 bp) were chosen as the seed sequence, with the rest aligned to form a contiguous sequence using SMRT portal assembly software (v5.0.1) [30]. The initial assembly outcome was refined using the variant Caller module from the SMRT Link software (v5.0.1) [30] and then polished with Illumina reads aligned by the Burrows–Wheeler Aligner (BWA) (v0.7.12) [31]. Using CheckM (Version 1.1.3) [32], the genome’s authenticity was validated, and the 16S rRNA gene sequence was compared to confirm its authenticity [33]. The NCBI Prokaryotic Genome Annotation Pipeline (PGAP) was utilized to predict genome components and gene functions [34]. Annotation details were visualized on circular genome maps created with Circos [35]. Functional annotations were performed using 6 databases: KEGG (Kyoto Encyclopedia of Genes and Genomes), COG (Clusters of Orthologous Groups), NR (Non-Redundant Protein Database), Swiss-Prot, GO (Gene Ontology), and TrEMBL. Furthermore, virulence factors were anticipated using the pathogen–host interactions database (PHI) and the virulence factors of pathogenic bacteria database (VFDB) [36,37]. The analysis of antibiotic resistance genes was conducted using the antibiotic resistance genes database (ARDB) and the comprehensive antibiotic research database (CARD) [38,39].

### 2.6. In Vitro Cytotoxicity-Test

DQ-SS1 were cultured in DMEM media to an OD600 ranging between 0.8 and 1.0 and centrifuged, and the cellular supernatants were collected and filtrated through a 0.22 µm bacterial filter. RAW 264.7 cells were grown in DMEM medium with the addition of 10% fetal calf serum and 1% mycillin. Prior to the experiment, RAW 264.7 cells were passaged and cultivated in a 12-well cell culture plate (10^6^ cells/well and supplied with a cell slide). The next day, the cellular supernatants were discarded, and 900 Ll fresh DMEM was added with either 100 µL of DQ-SS1cellular supernatants or 100 µL of DMEM (control). At 20 min, 40 min, and 60 min following incubation, the cell slides were stained with 0.4% trypan blue and visualized under a microscope (Leica DM1000, Hamburg, Germany). This assay was performed in duplicate.

### 2.7. Determination of Extracellular Enzymes and Hemolytic Effect

The extracellular enzymatic activities, including lipase, gelatinase, caseinase, amylase, and hemolysin of DQ-SS1, were analyzed as described in an earlier study [40,41]. In brief, DQ-SS1 and *E. coli* DH5α were grown in 2216E media until they reached an OD600 of 0.8; subsequently, the cells were washed and re-suspended in phosphate-buffered saline (PBS) to a concentration of 1 × 10^8^ CFU/mL. The 2216E agar plates were prepared by supplementing with 1% tween-80, 1% gelatin, 0.3% casein, or 1% starch. In addition, a hemolytic assay was conducted using a sheep blood agar plate (HopeBio, China). The extracellular enzyme and hemolytic activity assays were performed by introducing 100 μL of bacterial suspension into Oxford cups placed on the respective plates, which were incubated at 28 °C for 24 h and observed for the formation of halos. This assay was performed in duplicate.

### 2.8. Survival Assay

DQ-SS1, *Escherichia coli* DH5α, and *Edwardsiella tarda* TX1 [42] were cultured in 2216E media to an OD600 of 0.8, following which the bacterial cells were washed and re-suspended in PBS to achieve a concentration of 1 × 10^6^ CFU/mL. The serum was collected from healthy *S. schlegelii*, filtered through a 0.22 µm bacterial preof filter, and diluted to 1/2, 1/4, and 1/8. The bacterial cells were mixed with an equivalent volume of the undiluted serum, diluted serum, and PBS. After incubation at 28 °C for 1 h, the mixtures were further diluted, and colony-forming units (CFU) were quantified on 2216E agar plates. This assay was performed in duplicate.

### 2.9. In Vivo Experimental Infection Test

#### 2.9.1. Experimental Fish

Clinically healthy *S. schlegelii* (average body weight of 35.4 g and average length of 14.0 cm) were purchased from a commercial fish farm (Yantai, China) and housed in aerated tanks containing freshly prepared artificial seawater (changed daily) with a salinity of 30‰ and a temperature of 20 °C. Prior to any experiments, the fish were acclimated in the lab for two weeks and confirmed to be free from pathogenic bacteria. For tissue collection, the fish were euthanized with an overdose of tricaine methanesulfonate (Sigma, St. Louis, MO, USA).

#### 2.9.2. Infection and Tissue Distribution of DQ-SS1

For in vivo infection, DQ-SS1 was cultured in 2216E medium to an OD_600_ of 0.8, following which bacterial cells were washed and re-suspended in PBS to yield suspensions with varying concentrations ranging from 1 × 10^4^ to 1 × 10^8^ CFU/mL. Sixty healthy juvenile *S. schlegelii* were randomly assigned to eight groups (groups 1 to 6) and received either an intraperitoneal (i.p.) injection of 100 μL of DQ-SS1 suspensions or the same volume of PBS as a control and maintained at 22 °C. The physical condition of the infected fish was monitored every six hours, focusing on parameters such as feeding activity and swimming behavior. Moribund fish were euthanized using an overdose of tricaine methanesulfonate (Sigma, St. Louis, MO, USA) to minimize suffering. Cumulative mortality was recorded for two weeks. Bacteria were recovered from the freshly dead fish, and isolates were verified by 16S rDNA sequencing. The median lethal dose (LD_50_) was calculated using Karber’s method [43].

Regarding tissue distribution analysis, strain DQ-SS1 was cultured as described above and re-suspended in PBS to achieve a concentration of 10 × LD_50_. Ten healthy juvenile *S. schlegelii* were assigned to two groups and i.p. injected with either 100 μL of DQ-SS1 or PBS, respectively, and maintained at 22 °C. At 36 h post-injection (hpi), five fish were euthanized, and tissues from the liver, spleen, kidney, brain, gill, intestine, and muscle were collected and homogenized under aseptic conditions. The bacterial loads were determined using the TaqMan qPCR method with the Pro Taq HS Premix Probe real-time PCR Kit III (Accurate Biotechnology Co., Ltd., Changsha, China) [44]. These experiments were performed three times.

#### 2.9.3. Histopathology Analysis

*S. schlegelii* were cultured and challenged with DQ-SS1 as described above. Moribund fish were euthanized using an overdose of tricaine methanesulfonate. Subsequently, the liver, spleen, kidney, brain, gill, and intestine tissues were aseptically harvested from infected and control fish, and each tissue sample was divided into two parts. One portion was trimmed to the appropriate size and promptly preserved in 10% (*v*/*v*) neutral buffered formalin for a minimum of 24 h, while the rest was used for plate cultivation and 16S rRNA gene sequencing to verify that DQ-SS1 infection was the primary etiological agent for the disease. Then, the fixed specimens were rinsed, dehydrated, rendered transparent, infiltrated with wax, and embedded in paraffin to prepare tissue sections. Serial sections of 5 μm thick paraffin-embedded tissues were sectioned using a Leica™ microtome. Paraffin strips were spread in warm water at 37 °C and transferred onto slides, which were dried in a 37 °C oven. Ultimately, the sections were stained with hematoxylin and eosin (HE) for the examination of tissue pathology using a light microscope.

### 2.10. Statistical Analysis

The normality and homogeneity of the data were verified using SPSS 23 software (SPSS Inc., Chicago, IL, USA). Following verification, statistical significance was assessed using a one-way analysis of variance (ANOVA), followed by a post hoc Tukey test (pairwise comparisons). Survival rates were estimated using the Kaplan–Meier method and compared using the log-rank test. Statistical significance was set at *p* < 0.05.

## 3. Results

### 3.1. Clinical Symptoms of Naturally Infected Fish

The clinical manifestations observed in most naturally infected *S. schlegelii* included anorexia, lethargy, and punctate ulcerations on the body surface (Figure 1B). Necropsy of the affected fish revealed characteristic clinical signs, including mild ascites in the abdominal cavity, hemorrhaging and congestion in the liver, intestines, and jejunum, and swelling of the kidneys (Figure 1C).

### 3.2. Isolation and Identification of the Pathogen

**Figure 2:**
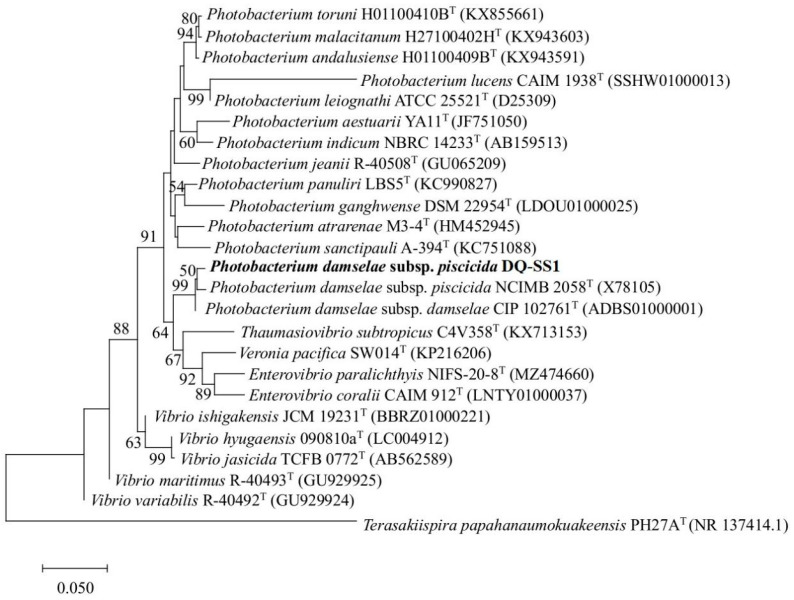
Neighbor-joining phylogenetic tree based on 16S rRNA gene sequences showing the phylogenetic positions of *Photobacterium damselae* subsp. *piscicida* DQ-SS1. Bootstrap values > 50% based on 1000 replications are presented at branching points. Scale bar, 0.05 substitutions per nucleotide position. Bold name, the bacterial strain analyzed in this study.

Following a 24 h incubation of the diseased fish tissue homogenate at 28 °C, circular, marginally convex, smooth, and ivory-white bacterial colonies were observed on the plate (Figure 1D). After three successive transfers to new 2216E agar plates, a pure bacterial culture was isolated and named DQ-SS1 (Figure 1E). The bacterial isolate was identified via 16S rDNA gene sequencing and submitted to Genebank (Accession number PQ891937). The phylogenetic tree analysis indicated that strain DQ-SS1 was grouped with recognized species of *P. damselae* subsp. *piscicida* and was closely associated with strain *P. damselae* subsp. *piscicida* NCIMB 2058^T^ (100% similarity) (Figure 2).

### 3.3. Biochemical Characteristics

The biochemical characterizations of strain DQ-SS1 were determined using the API ZYM, API 20NE, and API 50CH systems. The results of API 20NE showed that DQ-SS1 could utilize potassium nitrate, dextrose, l-arginine, urea, aescin iron citrate, dextrose, d-mannose, N-acetyl glucosamine, d-maltose, and malic acid. Meanwhile, the enzymatic profile acquired from API ZYM uncovered positive activity for alkaline phosphatase, leucine arylamidase, acid phosphatase, *α*-glucosidase, N-acetyl-*β*-glucosaminidase, oxidase, and catalase. Lastly, the API 50CH fermentation profile demonstrated that DQ-SS1 could generate acid from mannitol, d-ribose, d-galactose, d-glucose, d-fructose, d-mannose, N-acetyl-d-glucosamine, d-cellobiose, d-maltose, d-trehalose, starch, and glycogen (Table S1).

### 3.4. Antibiotic Sensitivity

To evaluate the antibiotic sensitivity of strain DQ-SS1, the disc diffusion method was used on 2216E agar plates and the inhibition zones around each disc were measured, which were categorized as resistant (R), intermediate (I), or sensitive (S). The results indicated that strain DQ-SS1 was resistant to 11 antibiotics, namely clindamycin, vancomycin, midecamycin, erythromycin, neomycin, gentamicin, amikacin, cefamezin, carbenicillin, oxacillin, and penicillin. At the same time, it was sensitive to 15 antibiotics, namely chloramphenicol, furazolidone, sulfamethoxazole, ciprofloxacin, ofloxacin, norfloxacin, doxycycline, tetracycline, cefoperazone, ceftriaxone, ceftazidime, cefradine, cephalexin, piperacillin, and ampicillin. Finally, it displayed intermediate sensitivity for four antibiotics, namely polymyxin B, minocycline, kanamycin, and cefuroxime (Table 1).

### 3.5. Genomic Analysis

**Figure 3:**
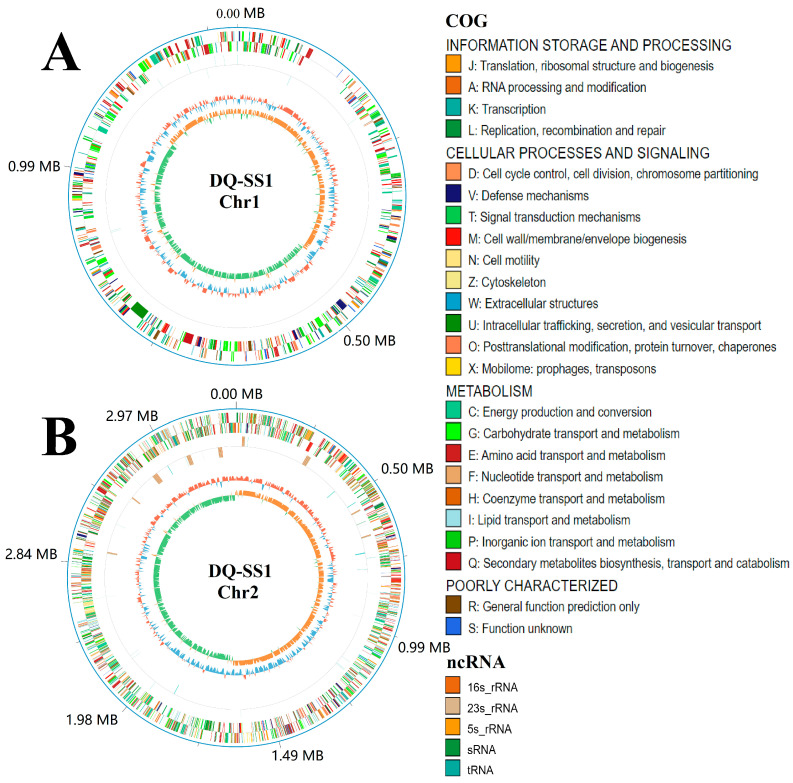
Circular maps of *Photobacterium damselae* subsp. *piscicida* DQ-SS1 chromosomes 1 (**A**) and 2 (**B**). The base pairs are indicated outside the outer circle (circle 1). Circles 2 to 5 represent coding genes, COG annotation, noncoding RNA (ncRNA), and GC content, respectively.

The genomic analysis of DQ-SS1 revealed two circular chromosomes with sizes of 1,273,877 bp and 3,236,568 bp (Figure 3), and no plasmids were found. The predicted counts for 5S, 16S, 23S rRNA, and tRNA sequences were 21, 19, 19, and 203, respectively (Table 2). Furthermore, the genome of DQ-SS1 revealed 8 genomics islands, 9 prophages, 2 CRISPRs, and 286 secreted proteins (Table 2). The COG category distributions for DQ-SS1 strains are provided in Table S2.

### 3.6. Virulence Factors and Antibiotic Resistance Genes Analysis

**Figure 4:**
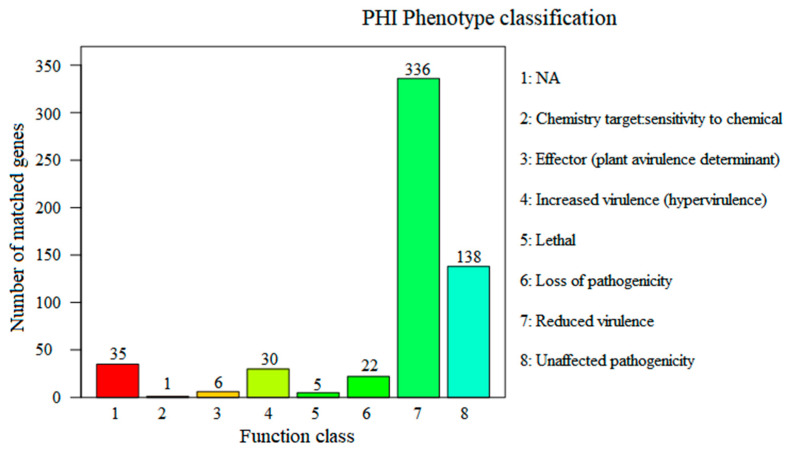
Pathogen–host interaction (PHI) annotation of the functional genes of DQ-SS1. Numbers above each column indicate the gene numbers of different pathogen PHI phenotypic mutant types. Different colors indicated different PHI phenotype classifications.

Functional genomic analysis was further performed to identify potential genes in DQ-SS1 associated with pathogenicity. PHI and VFDB analysis identified 573 and 314 genes related to pathogen-host interaction and virulence, respectively. Additional classification analysis with the PHI phenotype classification system showed that most genes related to pathogen–host interactions could be grouped into reduced virulence (336 genes), loss of pathogenicity (22 genes), and increased virulence (30 genes) (Figure 4). VFDB analysis further revealed that the virulence genes were involved in motility, adherence, and biofilm formation (flagella, MSHA pili, type IV pili, LOS, and LPS), the secretory system (T2SS, T4SS, and T6SS), immune escape, and metabolic regulation.

CARD database analysis identified only five genes associated with antibiotic resistance, of which four genes were associated with antibiotic efflux and one gene was associated with antibiotic target alteration. More importantly, ADRB analysis identified 21 genes associated with antibiotic resistance, largely targeting antibiotics such as penicillin, fluoroquinolone, carbenicillin, aminoglycoside, macrolide, streptomycin, kanamycin, and trimethoprim.

### 3.7. In Vitro Cytotoxicity

**Figure 5:**
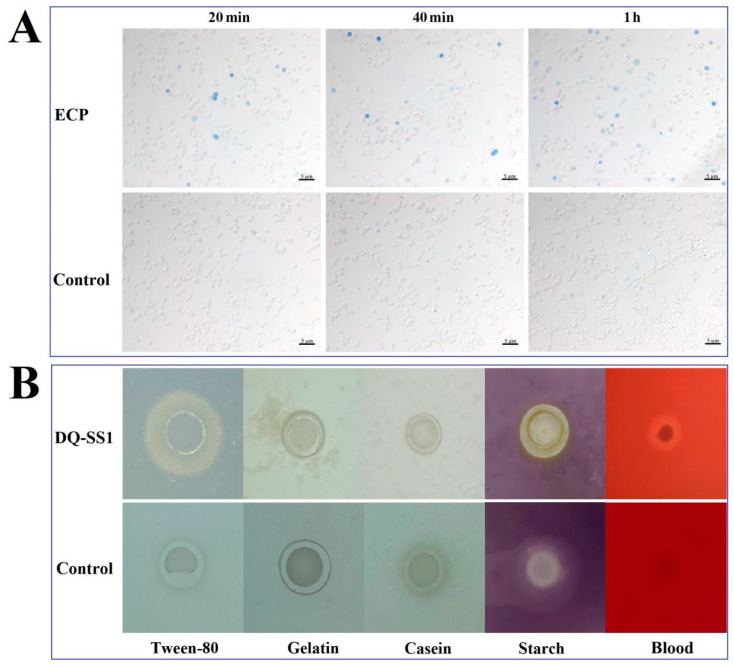
Expression of extracellular products (ECP) by DQ-SS1. (**A**) The in vitro cytotoxicity of ECP was analyzed in RAW 264.7 cells at different time points. (**B**) Extracellular hemolysin and lytic enzyme activities, including caseinase, lipase, amylase, and gelatinase, were analyzed. Control: *Escherichia coli* DH5α.

In order to determine the in vitro cytotoxicity of DQ-SS1 ECPs, the DQ-SS1 ECP was collected and introduced into RAW 264.7 cells. After staining with trypan blue, dead cells were visible as early as 20 min, with the proportion of dead cells increasing from 5.1% to 20.6% over incubation time (Figure 5A).

### 3.8. Determination of Extracellular Enzymes and Hemolytic Activities

The hemolytic activity and the presence of lytic enzymes, including lipase, gelatinase, caseinase, and amylase in strain DQ-SS1, were examined using 2216E agar plates with the appropriate substrates. The results of the hemolytic assay suggested that DQ-SS1 exerted hemolytic effects on sheep blood and formed a transparent hemolytic zone on the blood plate, reflecting *β*-hemolysis. The results of lytic enzyme activity assays showed that DQ-SS1 secreted amylase and lipase but did not secrete gelatinase and caseinase (Figure 5B).

### 3.9. Survival Assay

**Figure 6:**
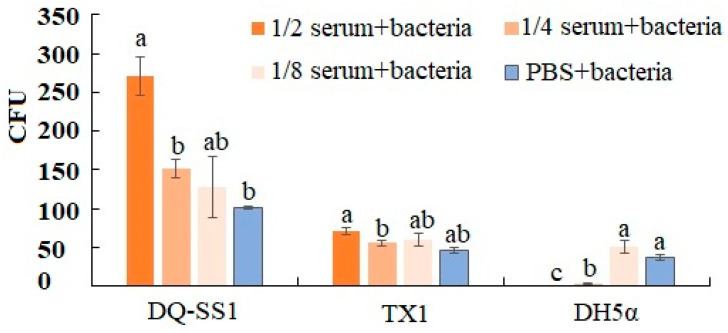
Bactericidal activity of *Sebastes schlegelii* serum against bacteria. Different concentrations of *Sebastes schlegelii* serum were incubated with *Photobacterium damselae* subsp. *piscicida* DQ-SS1, *Escherichia coli* DH5α, *Edwardsiella tarda* TX1, or PBS, and colony-forming units (CFUs) were calculated on 2216E agar plates. Values are expressed as means ± SE (*n* = 3). Different letters denote significant differences (*p* < 0.05) between samples.

In order to determine the survival ability of DQ-SS1 in the serum of *S. schlegeli*, a bactericidal assay was performed using *E. coli* DH5α and *E. tarda* TX1 (which can survive in fish serum) as positive and negative controls, respectively. The results showed that DQ-SS1 could not only survive in high-concentration serum but also significantly proliferate in the serum. In contrast, the control bacteria, *E. coli* DH5α, was significantly killed in the 1/4 and 1/2 serum dilutions, whereas *E. tarda* TX1 survived and proliferated in the serum (Figure 6).

### 3.10. Pathogenicity of P. damselae subsp. piscicida DQ-SS1

**Figure 7:**
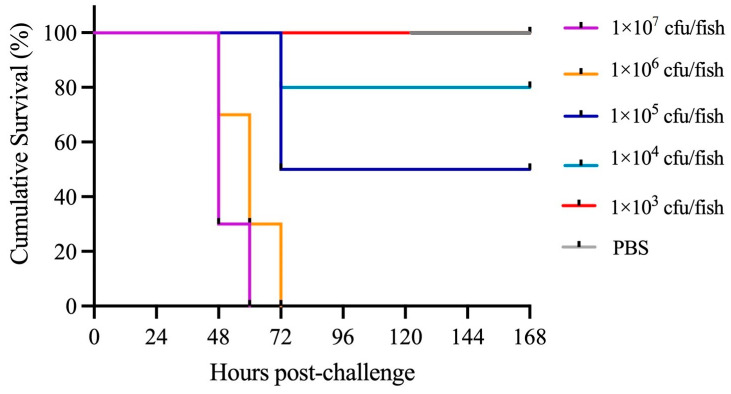
Cumulative survival of *Sebastes schlegelii* following *Photobacterium damselae* subsp. *piscicida* DQ-SS1 infection. *S. schlegelii* were challenged with varying concentrations of DQ-SS1 or PBS (Control), and the mortality was monitored every 12 h.

**Figure 8:**
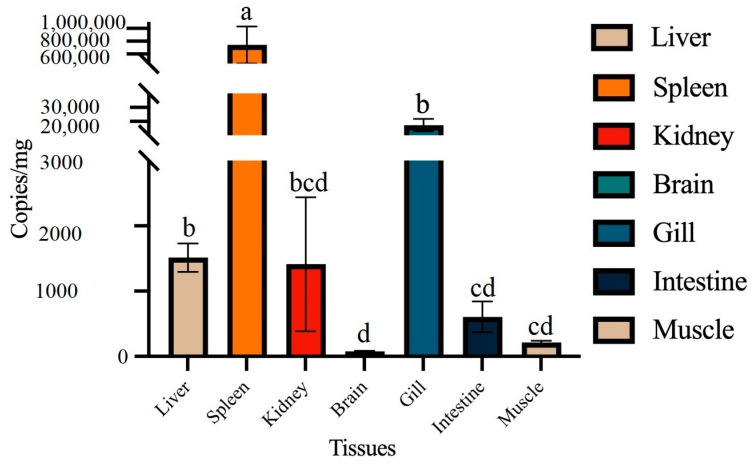
Tissue distribution of *Photobacterium damselae* subsp. *piscicida* DQ-SS1 in infected *Sebastes schlegelii*. *S. schlegelii* were artificially challenged with DQ-SS1, and the bacterial loads of DQ-SS1 in the liver, spleen, kidney, brain, gills, intestine, and muscles were determined. Values are presented as means ± SE (*n* = 3). Different letters represent significant differences (*p* < 0.05) between samples.

The in vivo infection assay indicated that the mortality of DQ-SS1-infected *S. schlegeli* was evident within 48 h and peaked within 72 h. On the other hand, no further clinical symptoms or mortality were noted after the 72 h mark. Likewise, no clinical symptoms or mortality were observed in the control group. Moreover, *P. damselae* subsp. *piscicida* DQ-SS1 was re-isolated from artificially infected *S. schlegelii* and confirmed as the original pathogen using phenotypic and molecular methods. The pathogenicity study unveiled mortality rates of 100%, 100%, 50%, 20%, and 0% in DQ-SS1-infected fish at concentrations of 1.0 × 10^7^, 1.0 × 10^6^, 1.0 × 10^5^, 1.0 × 10^4^, and 1.0 × 10^3^ CFUs, respectively, within 14 days (Figure 7). Moreover, the LD_50_ of DQ-SS1 to *S. schlegelii* was calculated to be 1.7 × 10^3^ CFU/g. Tissue infection and distribution analysis showed that DQ-SS1 was capable of infecting various tissues, including the liver, spleen, kidney, brain, gills, intestine, and muscles. Notably, the spleen exhibited the highest susceptibility to infection (Figure 8).

### 3.11. Pathological Analysis of Infected Fish

**Figure 9:**
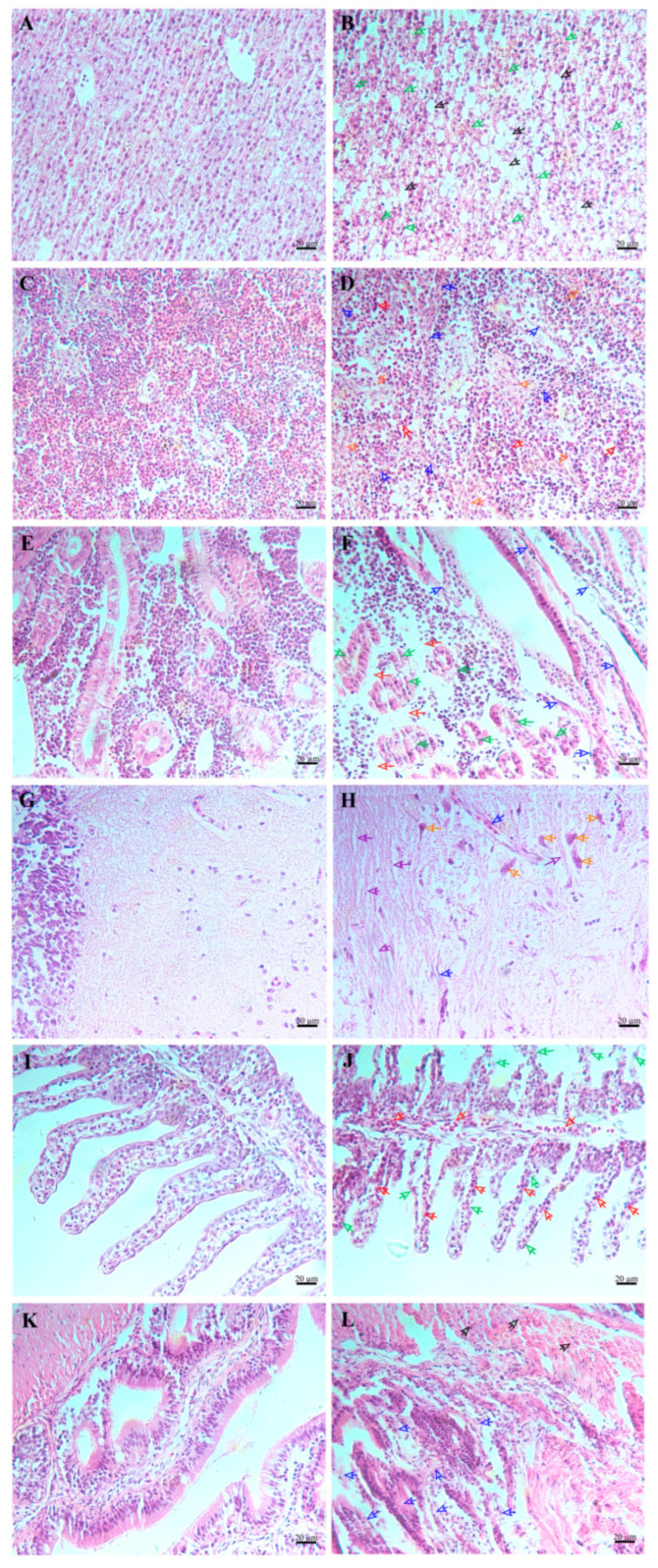
Histological changes in *Sebastes schlegelii* following *Photobacterium damselae* subsp. *piscicida* DQ-SS1 infection. (**A**,**C**,**E**,**G**,**I**,**K**) Histological sections of healthy liver, spleen, kidney, brain, gills, and intestine; (**B**,**D**,**F**,**H**,**J**,**L**) Histological sections of DQ-SS1 infected liver, spleen, kidney, brain, gills, intestine, and muscles. Scale bar = 20 μm.

Compared to the healthy fish (Figure 9A,C,E,G,I,K), significant histopathological alterations were noted in DQ-SS1-infected *S. schlegelii*. For example, the cell boundaries were blurred and disordered in the liver, featured by nuclear fragmentation, as well as hepatocyte necrosis, dissolution (indicated by green arrow), and vacuolation (Figure 9B, black arrow). In the spleen, inflammatory cell infiltration (red arrow), fibroblast proliferation (blue arrow), and nuclear fragmentation and dissolution were observed (Figure 9D, orange arrow). In the kidney, fibroblast proliferation (blue arrow) and severe necrotic foci were detected (green arrow), accompanied by vacuolar degeneration (Figure 9F, red arrow). In the brain, the dissolution of the tigroid body in the central region (orange arrow), fibroblast proliferation (blue arrow), and loose glial cell aggregation were noted (Figure 9H, purple arrow). In the gills, the tissue boundary was destroyed, with disrupted gill filaments (green arrow) and inflammatory cell infiltration (Figure 9J, red arrow). In the intestine, the structure of the intestinal mucosal layer and lamina propria disappeared, and the intestinal villi were atrophied, blurred, or absent (blue arrow) (Figure 9L, black arrow).

## 4. Discussion

*P. damselae* subsp. *piscicida* is recognized as the causative agent of pasteurellosis, which can infect different species of marine fish. During the rainy season, this disease is often observed, characterized by considerable rainfall, pronounced salinity shifts, and elevated temperatures in the water (over 20 °C) [45]. In August 2023, a sudden outbreak of disease attributed to PDP was documented in *S. schlegelii*, coinciding with environmental temperatures exceeding 24 °C, as well as swelling of the kidneys. To the best of our knowledge, this is the first study to report a natural PDP infection in cage-cultured *S. schlegelii*. Of note, the clinical signs of infection vary across fish species. Some fish, such as *Cynoglossus semilaevis*, exhibit hemorrhages and ulcerative lesions on the skin [15], whereas other species, such as cobia and golden pompano, show scattered nodules on the body surface or internal organs [16,17]. Pronounced ulceration on the body surface, along with liver and intestinal hemorrhage and congestion, was observed in diseased *S. schlegelii*. However, nodules were absent on the body surface and organs of diseased *S. schlegelii*, consistent with the findings of a previous study on PDP-infected *Lateolabrax maculatus* [46]. Taken together, these findings signal that the clinical signs of PDP infection may vary depending on the different fish species and PDP isolates.

In aquaculture, PDP is known as a highly dangerous bacterial pathogen due to its capability to infect numerous fish species and cause significant mortality [47]. Previous studies have established that extracellular products (ECPs), such as cytotoxins, protease, caseinase, lipase, and hemolysins, are the primary virulence factors of PDP and play a decisive role in bacterial infection and spread [45,46,47]. Indeed, pathogen-generated ECPs may increase vascular permeability at the infection site, causing edema and hemorrhage and inducing blood cell lysis, eventually resulting in the release of hemoglobin [17]. Cytolytic necrosis was observed in the liver, spleen, kidney, and gills during pathological examination, which might be caused by the release of bacterial toxins such as hemolysin or other extracellular enzymes. Comparable histological changes have also been reported in other fish species infected with PDP, *Aeromonas hydrophila*, or *V. harveyi* [48,49,50]. Herein, the cytotoxicity test demonstrated that the ECPs of PDP exhibited high virulence to RAW cells in a time-dependent manner. Furthermore, the DQ-SS1 strain exhibited extracellular activities, including *β*-hemolysin, lipase, amylase, urease, oxidase, catalase, and other extracellular enzymes, which may have facilitated the cytolytic necrosis of cells and organs.

Antibiotics remain a cost-effective and efficient approach for the management of bacterial pathogens and are extensively utilized in numerous countries. Nonetheless, their extensive application in different environments has resulted in the emergence and spread of different antibiotic-resistance genes (ARG) within the microbiota [51]. The widespread occurrence of antimicrobial resistance can be attributed to several factors, including the horizontal transfer of antibiotic-resistance genes, biofilm formation, gene mutation, and latent gene activation [52,53]. In the present study, DQ-SS1 was resistant to clindamycin, vancomycin, midecamycin, erythromycin, neomycin, gentamicin, amikacin, cefamezin, carbenicillin, oxacillin, and penicillin and intermediately resistant to polymyxin B, minocycline, kanamycin, and cefuroxime. To elucidate the underlying mechanisms, genomic analysis based on the CARD and ADRB databases was also performed. CARD analysis identified genes associated with antibiotic efflux and antibiotic target alteration, whilst ADRB analysis identified genes targeting antibiotics such as penicillin, fluoroquinolone, carbenicillin, aminoglycoside, macrolide, streptomycin, kanamycin, and trimethoprim [38,39]. Noteworthily, the drug-resistant spectrum of DQ-SS1 was comparable to that of the *P. damselae* subsp. *piscicida* NH-LM2 isolated from *L. maculatus* cultured in the same sea area [46]. Interestingly, NH-LM2 harbors three plasmids that encode seven genes linked to antibiotic inactivation. However, DQ-SS1 possesses no plasmids. Another research study investigating PDP isolated from Mediterranean Sea bass identified a different drug-resistant spectrum compared with DQ-SS1 and NH-LM2 [48]. Moreover, resistance against chloramphenicol, tetracycline, and ampicillin, which have been documented in the R plasmids of PDP, was not detected in strain DQ-SS1 [54,55,56]. However, resistance against erythromycin and kanamycin, identified in R plasmids, was present in strain DQ-SS1 [57,58]. The pervasive use of antibiotics across diverse geographical regions and sources exacerbates antimicrobial resistance [59]. Furthermore, non-antibiotic agents present in aquatic environments, such as metals, biocides, plant protection products, and non-antibiotic pharmaceuticals, significantly contribute to the variability of antibiotic resistance [60]. Collectively, these findings suggest that geographic distribution and environmental factors are crucial determinants in shaping the drug-resistant profiles of different strains. In addition, the expansion of the antimicrobial resistance spectrum should be paid more attention and stricter medication regulations should be published, along with the exploration of aquatic vaccines and probiotics.

Understanding the occurrence and pathogenicity of bacterial pathogens is essential for managing the health and ensuring the success of farmed fish. The LD_50_ metric is commonly employed to evaluate the pathogenicity of these bacterial pathogens. However, data on the pathogenicity of PDP toward *S. schlegelii* are limited. PDP is a harmful bacterial pathogen for marine fish due to its broad host range and high mortality rate [47]. Wang et al. documented an LD_50_ value of 1.1 × 10^6^ CFU/g of PDP for *T. ovatus* [16], whereas Liu et al. reported an LD_50_ value of 1.0^3^ × 10^4^ CFU/g of PDP for *R. canadum* [17]. Turbot was found to be highly susceptible to PDP with an LD_50_ of ≤1.6 × 10^4^ CFU/fish [61]. In this research, DQ-SS1 showed notable significant pathogenic effects on *S. schlegelii*, demonstrated by an LD_50_ value of 1.7 × 10^3^ CFU/g. Furthermore, mortality was observed within 48 h following exposure to DQ-SS1, suggesting a short incubation period. A comparable occurrence was observed in tongue soles infected with *Shewanella algae* and *Carassius auratus* infected with *A. veronii*, suggesting that a brief incubation period aids in the swift advancement of the disease [52,62].

To identify potential virulence genes, genomic analysis was performed based on the PHI and VFDB databases, which yielded 573 and 314 genes related to pathogen–host interaction and virulence, respectively [36,37]. Moreover, virulence genes involved in motility, adherence, secretory system, and immune escape were identified. The secretory system plays a vital role in the pathogenicity of pathogens that secrete various virulence factors. Herein, T2SS-, T4SS-, and T6SS-related genes were detected in PDP. However, T3SS was not detected. Several toxins, such as AIP56 and PnpA, have been reported to be secreted by T2SS and contribute to infection [19,20]. In 2019, while T3SS was identified in PDP [22], further investigation suggested that it might be encoded within an unstable plasmid [63], thus accounting for its absence in DQ-SS1. Complement is an integral component of the innate immune system that plays an instrumental role in the defense against bacterial pathogens in fish [64]. Some bacterial pathogens have evolved distinct mechanisms to escape complement-mediated killing [65,66]. Previous studies determined that only the classical complement activation pathway can kill PDP, whereas the other complement pathways exert minimal effects on PDP [24]. In this study, PDP was not sensitive to the normal serum of *S. schlegeli* and even proliferated in the serum, thereby promoting its survival and spread in the bloodstream.

## 5. Conclusions

This study characterized the pathogenic and genomic properties of PDP strain DQ-SS1 associated with ulceration in *S. schlegelii*. The genome of DQ-SS1 comprises two chromosomes and no plasmids. Moreover, a significant number of genes associated with antibiotic resistance and virulence were detected. DQ-SS1 exhibited significantly high pathogenicity towards *S. schlegelii*, with an LD_50_ value of 1.7 × 10^3^ CFU/g. Furthermore, the pathological alterations, cytotoxicity, and serum survival of the isolate were examined. However, this study has certain limitations, such as the inability of artificial infection to accurately mimic natural infection routes and environmental conditions, and the fact that a single PDP isolate cannot represent the epidemiological characteristics of PDP within this geographical region. Future research should focus on the epidemiological features, genetic variations, and vaccine trials related to PDP. Overall, this study provides valuable insights for a more comprehensive understanding of the pathogenic mechanisms of PDP and contributes to potential applications in (i) advancing diagnostic and preventative measures, (ii) highlighting the need for alternative treatments due to multi-antibiotic resistance, and (iii) identifying the potential threat of PDP to cage-cultured marine fish species, beyond just *S. schlegelii*.

## Figures and Tables

**Table 1:** Antibiotic susceptibilities of DQ-SS1.

Antibiotics	Concentration (µg per Disc Unless Otherwise Stated)	DQ-SS1
Clindamycin	2	R ^a^
Chloramphenicol	30	S
Furazolidone	300	S
Polymyxin B	300 IU	I
Sulfamethoxazole	1.25	S
Vancomycin	30	R
Ciprofloxacin	5	S
Ofloxacin	5	S
Norfloxacin	10	S
Midecamycin	30	R
Erythromycin	15	R
Minocycline	30	I
Doxycycline	30	S
Tetracycline	30	S
Neomycin	30	R
Kanamycin	30	I
Gentamicin	10	R
Amikacin	30	R
Cefoperazone	75	S
Ceftriaxone	30	S
Ceftazidime	30	S
Cefuroxime	30	I
Cefradine	30	S
Cefamezin	30	R
Cephalexin	30	S
Piperacillin	100	S
Carbenicillin	100	R
Ampicillin	10	S
Oxacillin	1	R
Penicillin	10 U	R

^a^ Abbreviations: I, intermediate; R, resistance; S, sensitive.

**Table 2:** Summary of the genome information of DQ-SS1.

Genome Feature	DQ-SS1
Genome size (bp)	4,510,445
Chr1 (bp)	1,273,877
Chr2 (bp)	3,236,568
Encoded genes	3923
Annotated genes	3843
5S rRNA	21
16S rRNA	19
23S rRNA	19
tRNA	203
sRNA	7
Genomics islands	8
Prophages	9
CRISPR	2
Secreted protein	286

## Data Availability

The original contributions presented in this study are included in the article/Supplementary Materials. Further inquiries can be directed to the corresponding author.

## Supplementary Materials

The following supporting information can be downloaded at: https://www.mdpi.com/article/10.3390/microorganisms13020441/s1, Table S1: Biochemical characteristics of *Photobacterium damselae* subsp. *piscicida* DQ-SS1.; Table S2: Clusters of Orthologous Group (COG) annotations of strain DQ-SS1 genome..
