# Supplementary material for: First Report and Pathogenicity Analysis of Photobacterium damselae subsp. piscicida in Cage-Cultured Black Rockfish (Sebastes schlegelii) Associated with Skin Ulcers
Source: Microorganisms. 2025 Feb 17;13(2):441. doi: 10.3390/microorganisms13020441 (PMC11858037; doi:10.3390/microorganisms13020441)
Supplement: Supplementary file 1 [file microorganisms-13-00441-s001.zip › microorganisms-3471630-supplementary.pdf]

Supplementary data

Table S1. Biochemical characteristics of *Photobacterium damselae* subsp. *piscicida* DQ-SS1.

Characteristic	DQ-SS1
**Utilize of**	
Potassium nitrate	+
L-tryptophan	-
Dextrose	+
L-Arginine	+
Urea	+
Aescin iron citrate	+
Gel (bovine source)	-
4-Nitrobenzene-β- D-galactopyranoside	-
Dextrose	+
L-arabinose	-
D-mannose	+
D-mannitol	-
N-Acetyl Glucosamine	+
D-maltose	+
Potassium Gluconate	-
Capric acid	-
Adipic acid	-
Malic acid	+
Sodium citrate	-
Phenylacetic acid	-
**Enzyme activity**	
Alkaline phosphatase	+
Esterase(C4)	-
Esterase lipase (C8)	-
Lipase (C14)	-
Leucine arylamidase	+
Valine arylamidase	-
Cystine arylamidase	-
Trypsin	-
Chymotrypsin	-
Acid phosphatase	+
Naphthol-AS-BI-phosphohydrolase	-
α-galactosidase	-
β-galactosidase	-
β-uronidase	-
α-glucosidase	+
β-glucosidase	-
N-acetyl-β-glucosaminidase	+
α-mannosidase	-
α-fucosidase	-
Oxidase	+
Catalase	+
**Acid from**	
Mannitol	+
Erythritol	-
D-arabinose	-
L-arabinose	-
D-ribose	+

D-xylose	-
L-xylose	-
D-side-marigold alcohol	-
Methyl-β- D-xylopyranoside	-
D-galactose	+
D-glucose	+
D-fructose	+
D-mannose	+
L-sorbose	-
L-rhamnose	-
Dulcitol	-
Inositol	-
Mannitol	-
Sorbitol	-
Methyl-α-D-mannopyranoside	-
Methyl-α-D-glucopyranoside	-
N-acetyl-D-glucosamine	+
Amygdalin	-
ARBULIN	-
Esculin ferric citrate	-
Salicin	-
D-cellobiose	+
D-maltose	+
D-lactose	-
D-melibiose	-
D-sucrose	-
D-trehalose	+
Inulin	-
D-melezitose	-
D-raffinose	-
Starch	+
Glycogen	+
Xylitol	-
D-gentiobiose	-
D-turanose	-
D-lyxose	-
D-tagatose	-
D-fucose	-
D-fucose	-
D-arabitol	-
D-arabitol	-
Gluconate	-
2-keto-gluconate	-
5-keto-gluconate	-

+, positive; –, negative.

Table S2 Clusters of Orthologous Group (COG) annotations of strain DQ-SS1 genome.

**Categories**	**Function**	**Gene number**	**Ratio (%)**
A	RNA processing and modification	1	0.03
C	Energy production and conversion	223	6.71
D	Cell cycle control, cell division, chromosome partitioning	55	1.65
E	Amino acid transport and metabolism	291	8.75
F	Nucleotide transport and metabolism	108	3.25
G	Carbohydrate transport and metabolism	204	6.14
H	Coenzyme transport and metabolism	200	6.02
I	Lipid transport and metabolism	116	3.49
J	Translation, ribosomal structure and biogenesis	259	7.79
K	Transcription	247	7.43
L	Replication, recombination and repair	133	4.00
M	Cell wall/membrane/envelope biogenesis	237	7.13
N	Cell motility	78	2.35
O	Posttranslational modification, protein turnover, chaperones	185	5.56
P	Inorganic ion transport and metabolism	182	5.47
Q	Secondary metabolites biosynthesis, transport and catabolism	35	1.05
R	General function prediction only	212	6.38
S	Function unknown	133	4.00
T	Signal transduction mechanisms	199	5.98
U	Intracellular trafficking, secretion, and vesicular transport	80	2.41
V	Defense mechanisms	100	3.01
W	Extracellular structures	31	0.93
X	Mobilome: prophages, transposons	14	0.42
Z	Cytoskeleton	2	0.06
